# Supplementary material for: Beyond Fish Oil Supplementation: The Effects of Alternative Plant Sources of Omega-3 Polyunsaturated Fatty Acids upon Lipid Indexes and Cardiometabolic Biomarkers—An Overview
Source: Nutrients. 2020 Oct 16;12(10):3159. doi: 10.3390/nu12103159 (PMC7602731; doi:10.3390/nu12103159)
Supplement: Supplementary file 1 [file nutrients-12-03159-s001.pdf]

Supplementary table 1. Identification of RCTs that investigated the effects of nuts and seeds rich in ALA through the database search.

**Study details**	**Study type**	**Number of participants**	**Population characteristics**	**Dose**	**Outcomes**
(Chisholm et al. 1998)	Randomised crossover study	21	Moderately hyperlipidaemic subjects	78g/d walnuts or another low-fat diet (fat 30% total energy)	TC and LDL were significantly lower, and HDL was significantly higher in both low-fat diets
(Domènech et al. 2019)	RCT	236	Older adults with mild hypertension	15% of habitual daily energy intake as walnuts or a control group consuming no nuts, for 2 years	A near significant (p=0.051) decrease in systolic BP was observed compared to control
(Fatahi et al. 2019)	Randomised controlled trial	99	Overweight and obese women	Either 300g/week of fish, 18 walnuts/week or 150g fish and 9 walnuts/week	The fish + walnut group experienced significantly higher reduction in SBP, fasting blood glucose, LDL, hs-CRP, TNF-alpha, IL6 and a significantly higher increase in HDL than the fish only and walnut only groups.<br><br>A significant decrease in TG and DBP was seen in the fish only and walnut only groups, compared to the fish + walnut group.

(Iwamoto et al. 2002)	RCT<br><br>(controlled, single-blind, cross-over study)	40	Young Japanese men and women	12.5% of energy derived from walnuts (44-58g/d) or a control diet (typical Japanese diet), for 4 weeks	TC and ratio of LDL to HDL was significantly lowered in the walnut group compared to the control, with LDL concentration was significantly lowered in women on the walnut diet but not men.<br><br>Women and men had a significant increase in blood ALA following walnut diet and this was correlated to the decreased LDL in women.
(Le et al. 2016)	RCT	213	Overweight and obese women	One of three diets for 6 months:<br><br>A walnut rich diet comprising 35% energy from fat and 45% energy from carbohydrates<br><br>A lower fat (20%), higher carbohydrate (65%) diet	TG were significantly decreased in all groups. HDL was increased significantly more in the walnut rich group than the low-fat or low-carbohydrate groups.<br><br>For insulin sensitive women, LDL was significantly reduced in the walnut group, whilst the low-fat group had significantly reduced TC and HDL.

				A higher fat (35%), lower carbohydrate (45%) diet	
(Machado et al. 2015)	Parallel, single-blind clinical trial	75	Overweight adolescents	28g/d brown flaxseed, golden flaxseed or the equivalent wheat bran 5 times per week for 11 weeks	Brown and golden flaxseed groups showed significant reduction in DBP. No significant differences between groups were observed for lipid profile or inflammatory markers, although all groups significantly increased levels of TNF-alpha
(Morgan et al. 2002)	Randomised, open-label crossover study	42	Patients with borderline high cholesterol	A low fat, low cholesterol diet for six weeks, followed by the same diet with 64g/d walnuts for six weeks	The walnut diet reduced TG significantly compared to baseline, whereas the non-walnut diet did not. There was a near significant (0.055) reduction in TC following walnut diet but not following the non-walnut diet.
(Muñoz et al. 2001)	Randomised, cross-over feeding trial	10	Men with polygenic hyper-cholesterolemia	A diet replacing ~35% energy with walnuts or a control diet (typical Mediterranean-type cholesterol-lowering diet)	Neither diet resulted in a significant change in TC, LDL, HDL or TG.
(Rock et al. 2017)	Within-subject crossover study	28	Obese adults	Either a meal containing 54%	Both meals significantly increased insulin at 60 mins

				energy from walnuts or a control (cream cheese)	but the walnut meal resulted in significantly reduced insulin afterwards at 120 minutes.
(Ros et al. 2004)	Randomised crossover study	18	Hyper-cholesterolemic subjects	A typical Mediterranean diet and a similar diet in which 32% of the MUFA energy is replaced by walnuts, both for 4 weeks	The walnut diet significantly reduced TC and LDL and these reductions were significantly associated with increased dietary ALA content.<br><br>There was no significant difference between the change in CRP after both diets.
(Sabaté et al. 1993)	Controlled, single-blind crossover study	18	Healthy male volunteers	Either a mixed, natural, cholesterol-lowering diet or a diet containing identical food and macronutrients, but 20% of the calories are derived from walnuts.	The walnut diet reduced TC, LDL, HDL and LDL:HDL ratio significantly more than the reference diet. Neither diet significantly altered TG or BP.
(Sanchis et al. 2019)	Randomised controlled crossover study	13	Volunteers with stage 3 or 4 chronic kidney disease.	A diet containing 30g/d of walnuts or a control diet, which was the same diet	The nut diet led to a significantly greater reduction in SBP but not DBP. The walnut diet significantly reduced LDL, but not

				without the walnuts, both for 30 days<br><br>*(control diet also contained 60g white bread and 5g olive oil per day in place of walnuts)*	significantly more than the control diet (p=0.077).
(Singh et al. 2002)	Randomised, single blind trial	1000	Patients with a presence or risk factor of CAD	A standard Mediterranean cholesterol-lowering diet or an intervention diet containing more than double the alpha-linolenic acid (1.8g/d vs 0.8g/d)	The ALA diet resulted in a significantly greater reduction in SBP, DBP, TC, LDL and TG than the reference diet. The ALA diet significantly increased HDL whereas the reference diet decreased HDL.
(Tapsell et al. 2004)	Parallel, randomised controlled trial	57	Healthy volunteers	One of three diets with 30% energy as fat. (1) a low-fat diet, (2) a low/modified fat diet, or (3) a low/modified fat diet with 30g/d walnuts.	The walnut group experienced a significantly greater increase in HDL than the other groups. LDL significantly decreased in the walnut group and did not change significantly in the other groups. No significant difference for TG between groups was observed.

(Tindall et al. 2019)	Randomised, controlled, 3-period crossover study	45	Individuals at risk for CVD	One of three isocaloric maintenance diets. (1) a walnut diet, where 18% energy was derived from walnut snacks, (2) a walnut fatty acid matched diet, which had the same fatty acid composition as (1) but no walnuts, and (3) a diet that replaced the ALA from walnuts with oleic acid.	There was no significant difference in the effect on central SBP between groups. The decrease in central SBP was greater in the walnut diet than in the oleic acid diet, but no differences between other combinations. All diets significantly reduced TC, LDL and HDL, but there were no significant differences between groups.
(Tuccinardi et al. 2019)	Crossover, randomised, double-blind, placebo-controlled trial	10	Obese individuals	48g/d walnuts or a macronutrient-matched, nut-free smoothie	Following the study, the walnut group had near-significantly (p=0.06) lower large VLDL and significantly higher medium VLDL and LDL than control.
(Wu et al. 2014)	Controlled, cross-over study	40	Healthy adult volunteers	43 g/d walnuts and then a control diet (or vice-versa)	The walnut diet significantly reduced non-HDL cholesterol compared to the control diet. No significant changes were

					found for LDL, HDL, TG, insulin, glycated haemoglobin, or CRP. TC was reduced near-significantly (p = 0.073) more by the walnut diet than the control.
(Zambón et al. 2000)	Randomised, crossover study	49	Adults with polygenic hyper-cholesterolemia	Either a Mediterranean diet or a similar diet with 35% energy from MUFA is replaced with walnuts	The walnut diet resulted lowered TC and LDL significantly more than the control diet.
(Zibaeenezhad, Shamsnia and Khorasani 2005)	Randomised case control study	52	Healthy volunteers	Group A consumed walnuts, 20 grams per day for 8 weeks and the control group (group B) consumed no walnuts.	The walnut group had significantly reduced TG and significantly increased HDL and no significant change in LDL or TC. The control group had no significant change in TG, HDL, LDL, or TC.

Supplementary table 2. Identification of RCTs that investigated the effects of ALA-rich oils through the database search.

Study details	Study type	Number of participants	Population characteristics	Dose	Outcomes
(Akrami et al. 2020)	RCT	52	Patients with metabolic syndrome	25ml/d flaxseed oil or a control (25ml/d sunflower oil)	Serum IL-6 was significantly reduced in the flaxseed oil group compared to the sunflower oil group.
(Barceló-Coblijn et al. 2008)	RCT	62	Firefighters<br><br>(An occupation traditionally exposed to CVD risk factors)	1.2, 2.4, or 3.6g/d flaxseed oil or 0.6 or 1.2g/d fish oil	No significant difference between changes to TC or HDL between groups.
(Freire et al. 2016)	Randomised, double blind clinical trial	52	Treated hepatitis C patients	6000mg/d FO or a soybean oil control	FO supplementation was associated with significantly greater reduction in serum insulin and glycated haemoglobin than the soybean oil. FO supplementation resulted in a significant increase in IL-1 and TNF-alpha and no significant change was seen for soybean oil.
(Ganji and Kies 1996)	Randomised crossover study	10	Normolipidemic humans	One of four diets: (1) soybean oil, (2)	No significant difference in TC, LDL, HDL, LDL:HDL ratio or TG

				soybean oil + psyllium husk, (3) corn oil, and (4) corn oil + psyllium husk<br><br>The soybean and corn oil diets consisted of breads containing 20% energy derived from soybean and corn oil respectively. Psyllium husk supplementation was added to these diets at 20g/d. Each diet was consumed for 7 days	were observed following soybean oil diet. Corn oil significantly increased TC, LDL, LDL:HDL ratio and TG and significantly decreased HDL. Addition of psyllium husk to soybean oil resulted in a significant decrease in TC, LDL and LDL:HDL ratio.
(Hamazaki et al. 1996)	RCT	24	Healthy, normolipidemic adults	Either a DHA-rich fish oil capsule or a control capsule containing 97% soybean oil	No significant differences were for TC, HDL, LDL or TG.
(Jamilian et al. 2020)	RCT<br><br>(randomised, double-blind,	60	Women with gestational diabetes	2000mg/d flaxseed oil (containing	LDL receptor was significantly upregulated and IL-1 and TNF-alpha were

	placebo-controlled trial)			800mg/d ALA) or a placebo	significantly down-regulated compared to placebo. Fasting glucose, insulin, insulin resistance, TC, TC:HDL ratio, VLDL and CRP were significantly decreased compared to placebo.
(Karupaiah et al. 2016)	Double-blind, randomised controlled cross-over study	34	Healthy adult volunteers	20g/d of soybean oil-based mayonnaise or palm olein-based mayonnaise	The soybean mayonnaise resulted in a significant reduction in TC, LDL and HDL compared to the palm-olein based mayonnaise, without affecting LDL:HDL ratio.
(Kaul et al. 2008)	RCT<br><br>(double blinded, placebo controlled, clinical trial)	86	Healthy adult volunteers	1g/d fish oil, flaxseed oil, hempseed oil or placebo	No significant changes in TC, HDL, LDL and TG were found for any of the groups.
(Kurowska et al. 1997)	Randomised 3-treatment crossover trial	34	Healthy adult volunteers with elevated LDL	Either 2% cow's milk, soybean or combination of milk and soybean products, incorporated into regular diet	Following the soybean diet, HDL was significantly increased and LDL:HDL ratio was significantly decreased. The response was associated initial LDL:HDL ratio, indicating that the

					response is more pronounced in individuals at higher risk of CAD.
(Layne et al. 1996)	Randomised, double-blind, controlled, cross-over study	26	Healthy volunteers	35mg/kg body weight per day of flaxseed oil or fish oil, for three months	TC, LDL and HDL were not significantly affected by either fish or flaxseed oil.<br><br>TG was significantly lower after fish oil supplementation in individuals with a low dietary PUFA:SFA (P/S) ratio, but not with a high PUFA:SFA ratio.
(Mirfatahi et al. 2016)	Randomised, double-blind clinical trial	34	Haemodialysis patients	6g/d flaxseed oil or 6g/d control (medium chain triglyceride oil)	Serum hs-CRP was significantly reduced following the flaxseed oil supplementation and this reduction was significantly greater than the control.
(Sierksma, Weststrate and Meijer 1999)	Double-blind crossover study	76	Healthy adult volunteers	Spreads fortified with soybean oil (0.8g/d) or shea nut oil (3.3g/d) compared to a non-fortified control spread, for 3 weeks each	The soybean oil spread significantly reduced LDL and TC levels compared to control but did not significantly affect HDL.

(Soleimani et al. 2017)	Parallel, randomised, double-blind, placebo-controlled study	60	Patients with diabetic nephropathy	1000mg/d flaxseed oil or placebo for 12 weeks	Serum insulin and TG were significantly decreased in the flaxseed group compared to placebo.<br><br>Significant decreases in insulin resistance, TC, LDL, and TC:HDL ratio were observed in the flaxseed group, but these were not significantly different to the control group.
(St-Onge et al. 2003)	Randomised crossover study	24	Healthy overweight men	Diets containing 40% energy as fat, 75% of which was added as flaxseed oil or olive oil.	TC was significantly decreased in the flaxseed oil group compared to the olive oil group. No significant change in LDL, HDL or TG was observed in either group.
(Thomsen et al. 2004)	Double-blind, randomised, placebo-controlled, three-arm crossover study	71	Mildly hyper-cholesterolemic patients	500ml milk blend with (1) 1.2g/d free plant sterols, (2) 1.6g/d free plant sterols, or (3) 0g soybean as a control, for four weeks each.	Both soybean containing groups reduced LDL significantly compared to control, however there was no significant difference in this reduction between soybean groups.

				Free plant sterols were predominantly from soybeans.	
(Utarwuthipong et al. 2009)	Randomised crossover study	16	Hyper-cholesterolemic women	A weight-maintaining diet supplemented with either (1) soybean oil, (2) rice bran oil, (3) palm oil, or (4) a rice bran oil/palm oil combination, for 10 weeks each.	TC and LDL were significantly reduced following diets (1), (3) and (4). HDL was significantly reduced following diet (1)
(Wallace, Miles and Calder 2003)	RCT<br><br>(randomised, placebo-controlled, double- blind, parallel study)	40	Healthy adult males	3.5g/d ALNA from flaxseed oil, 0.44, 0.94 or 1.9g/d EPA+DHA from FO, or placebo for 12 weeks	Neither intervention affected TNF-alpha. 0.94g and 1.9g doses of FO significantly decreased IL-6 production.
(Weststrate and Meijer 1998)	Randomised double-blind placebo-controlled incomplete Latin-square design	95	Healthy non-obese normo-cholesterolemic or mildly hyper-cholesterolemic	Non-hydrogenated margarine fortified with either soybean, shea nut or rice bran oil or sitostanol-ester (15-3.3g/d fortification) or a	TC and LDL were significantly reduced for soybean and sitostanol compared to control. No effects on HDL concentrations were observed.

				non-fortified placebo	
(Wilkinson et al. 2005)	RCT	57	Men with atherogenic lipoprotein phenotype	One of three diets supplemented with (1) flaxseed oil (0.5 n6:n3 ratio), (2) sunflower oil (27.9 n6:n3 ratio) or (3) sunflower oil with fish oil (5.2 n6:n3 ratio).	TC was significantly reduced by all diets, with diet (1) reducing significantly more. HDL was significantly reduced following (1) but not by the other diets. TG was significantly reduced by (1) and (3). No significant differences in LDL were observed.
(Yang et al. 2019)	RCT<br><br>(double-blind, randomized controlled trial)	108	Volunteers with abdominal obesity	2g/d FO (DHA + EPA), 2.5g/d flaxseed oil (ALA) or a corn oil control, for 12 weeks	FO significantly decreased LDL compared to CO, whereas flaxseed oil did not. No significant difference for any outcome was seen between FO and flaxseed oil groups.
(Zheng et al. 2016)	Double-blind, randomised controlled trial	185	Patients with type 2 diabetes	Fish oil (containing 2g/d EPA + DHA) or flaxseed oil (2.5g/d ALA) or a corn oil control	FO significantly reduced HbA1c compared to corn oil.<br><br>FO significantly decreased LDL, TC, TC:HDL and TG over time, whereas flaxseed oil had no significant

					effect on these blood lipids.
(Zibaeenezhad et al. 2003)	Randomised, double-blind, case-control study	60	Hyperlipidaemic individuals	3g/d walnut oil capsules or a placebo for 45 days	TG was significantly decreased n the walnut oil group compared to placebo. No significant change was observed for TC, LDL or HDL.
(Zibaeenezhad et al. 2016)	RCT	100	Patients with type 2 diabetes	15g/d walnut oil for 3 months or a control group with no intervention	Significant reduction in HbA1c was observed in the walnut oil group. No significant changes in SBP or DBP were observed in either group.
(Zibaeenezhad et al. 2017b)	Randomised, double-blind, placebo-controlled study	100	Hyperlipidaemic type 2 diabetes patients	15ml walnut oil or a placebo for 90 days	Significant decreases in TC, TG, LDL, and TC:HDL ratio were observed in the walnut group compared to placebo. A near significant ($p = 0.06$) increase in HDL in the walnut group compared to placebo.

Supplementary table 3. Identification of RCTs that investigated the effects of seaweed through the database search.

Study details	Study type	Number of participants	Population characteristics	Dose	Outcomes
(Ebrahimi-Mameghani et al. 2017)	RCT<br><br>(double-blind, placebo-controlled, randomised controlled trial)	70	Patients with non-alcoholic fatty liver disease	300mg/d *C. vulgaris* or a placebo for 8 weeks	There was a significantly greater decrease in TNF-alpha in the *C. vulgaris* group than the placebo. There were no significant differences between changes in insulin, insulin sensitivity and hs-CRP.
(Haidari et al. 2018)	Clinical trial	44	Women with primary dysmenorrhea	1500mg/d of Chlorella soft gels or placebo for 8 weeks	There was a significantly greater decrease in hs-CRP in the intervention group compared to placebo.
(Juárez-Oropeza et al. 2009)	Clinical trial	36	Healthy volunteers	4.5g/d *Spirulina maxima* for 6 weeks	Plasma TG, TC and LDL were significantly decreased, and HDL were significantly increased in the

					intervention group compared to placebo. Blood pressure was significantly decreased in the intervention group compared to placebo.
(Krotkiewski et al. 1991)	Double-blind crossover study	62	Middle-aged patients with mild hypertension	12-24g/d seaweed fibre or a placebo for 12 weeks	Mean blood pressure was significantly decreased by the seaweed fibre but not the placebo.
(Lee and Jeon 2015)	Randomised, double-blind, placebo-controlled trial with parallel group design	80	Pre-diabetic male and female adults	1500mg/d *E. cava* for 12 weeks compared to a placebo.	The *E. cava* group showed a significant decrease in insulin compared to the placebo.
(Martínez-Sámano et al. 2018)	Prospective, randomised, parallel pilot study	16	Patients with systemic arterial hypertension	4.5g/d Spirulina (*Arthrospira maxima)* or a placebo for 12 weeks	The *S. maxima* group had a significant decrease in systolic blood pressure compared to placebo.
(Mazokopakis et al. 2014)	Open-label, non-randomised study	52	Dislipedaemic volunteers	1g/d Spirulina (*Arthrospira plantesis)* for 12 weeks	Mean TG, LDL, TC and TC:HDL ratio were significantly decreased, whilst HDL was not

					significantly increased. No significant change in BP was observed.
(Mizoguchi et al. 2008)	Nutrigenomic study	17	Healthy subjects and subjects with high-risk factors for lifestyle diseases.	40 tablets/d which contain 95.5% *Chlorella*, for 16 weeks .	*Chlorella* intake resulted in significant reductions in TC, HDL and LDL in the high-risk group but not the healthy group at the end of the trial. After just 4 weeks, there were significant reductions in TC, HDL and LDL in both groups, but these reductions had returned to not significantly different to baseline levels by week 16.
(Paradis, Couture and Lamarche 2011)	Double-blind, randomized, placebo-controlled crossover study	23	Healthy volunteers	500mg/d seaweed (*Ascophyllum nodosum* and *Fucus vesiculosus*) or a placebo before	Insulin sensitivity was significantly increased when ingestion of 50g bread was accompanied by the seaweed

				consumption of 50g bread	capsule than the placebo.
(Torres-Durán et al. 2012)	Clinical trial	41	Healthy volunteer runners	5g/d Spirulina (*Arthrospira maxima)* powder for 15 days	Fasting TG levels significantly were lower after treatment with Spirulina
(Torres-Duran, Ferreira-Hermosillo and Juarez-Oropeza 2007)	Clinical trial	36	Healthy volunteers	4.5g/d Spirulina (*Arthrospira maxima*) for 6 weeks	There was a significant decrease TC and significant increase in HDL, but these changes were dependent on TG concentration. LDL and TG were significantly reduced independent of TG concentration.

Supplementary table 4. Identification of recent RCTs that investigated the effects of fish oil supplementation through the database search.

Study details	Study type	Number of participants	Population characteristics	Dose	Outcome
(Al-Ghannami et al. 2016)	RCT	314	Children aged 9 and 10 years	Fish oil (FO) capsule containing 403mg DHA and 53mg EPA daily for 12 weeks	No significant change in total lipids (cholesterol + triglycerides)
(Al-Ghannami et al. 2018)	RCT<br><br>(randomized double-blind, placebo-controlled trial)	285	Children aged 9 and 10 years	FO capsule containing 403mg DHA and 53mg EPA daily for 12 weeks	FO group had a significantly lower SBP and DBP compared to the fish meal group, but no significant difference in TC, HDL or LDL
(Alves Luzia et al. 2015)	RCT<br><br>(randomized double-blind, placebo-controlled trial)	59	Healthy women aged 40-70 in Brazil	FO capsule containing 540mg EPA and 360mg DHA twice a day for 3 months	No significant difference between groups for TC, TG, LDL or HDL were observed.
(Amini et al. 2018)	RCT<br><br>(randomized double-blind,	60	Women aged 18-40 with polycystic ovary syndrome	2 x 1g FO capsule daily for 12 weeks	Serum insulin levels were significantly decreased in the FO group and quantitative insulin sensitivity check index was significantly increased. High-

	placebo-controlled trial)				sensitivity CRP was significantly decreased.<br><br>No significant change in HDL, LDL, TC, TG or TC/HDL ratio was observed.
(Barbosa, Melo and Damasceno 2017)	RCT<br><br>(randomized double-blind, placebo-controlled trial)	80	Individuals with at least one cardiometabolic risk factor<br><br>(excess weight, hypertension, dyslipidaemia, diabetes or smoking)	3g/d FO (containing 37% EPA and 23% DHA) for 2 months	FO group showed significantly greater reduction in body fat compared to control.<br><br>FO group showed a significant reduction in TG, TC, LDL and HDL, but these reductions were not significantly greater than in the control group.
(Binia et al. 2017)	One-arm study	191	Healthy young Mexican adults	2.7g/d FO capsule (EPA and DHA) for 6 weeks	Mean fasting insulin and glycated haemoglobin were significantly decreased. No significant change was observed for TG, TC, LDL, HDL, CRP, IL-6 or expression of pro-inflammatory genes (*PPAR*) was observed.
(Cormier et al. 2016)	Clinical trial	191	Healthy volunteers	5g/d FO for 6 weeks	No significant change in CRP, TNF-alpha or IL-6 observed
(Cottin et al. 2016)	Single-blind, randomized, placebo-controlled, parallel study	48	Healthy young men	3g/d of DHA or EPA or a control of olive oil	No significant change in BP was observed for any groups
(Da Boit et al. 2017)	RCT	50	Healthy volunteers	3g/d FO for 18 weeks	A significantly greater decrease in TG was seen in the FO group

	(randomized double-blind, placebo-controlled trial)				compared to the placebo, no differences in BP, insulin, IL-6 or TNF-alpha were observed.
(Danthiir et al. 2018)	RCT<br><br>(double-blind, randomised, placebo-controlled, parallel-group trial)	390	Australian community dwelling adults (aged 65-90)	1720mg DHA and 600mg EPA daily for 18 months	The FO group showed a significant increase n HDL and decrease in triglycerides.
(Drudi et al. 2017)	RCT<br><br>(randomised, double-blinded, placebo-controlled trial)	80	Patients with peripheral arterial disease	4.4g/d FO capsules (2.6g EPA + 1.8g DHA)	Higher baseline LDL levels were associated with a more favourable response to FO treatment compared to placebo
(Franzese et al. 2015)	Observational case series study	600	Patients with suspected coronary artery disease	Median of 1200mg/d FO	FO supplementation was associated with significantly lower TG and VLDL in the whole population, but not in patients on lipid lowering therapy.<br><br>Patients not on lipid-lowering therapy had significantly lower VLDL, TG, and LDL when given FO supplementation.
(Freire et al. 2016)	Randomised, double blind clinical trial	52	Treated hepatitis C patients	6000mg/d FO	FO supplementation was associated with significantly reduced serum insulin and glycated haemoglobin

					and a significant increase in IL-1 and TNF-alpha
(Grenon et al. 2015)	RCT<br><br>(Randomised, double-blinded, placebo-controlled trial)	80	Patients with peripheral arterial disease	4.4g/d FO for 1 month<br><br>(2.6g EPA + 1.8g DHA)	FO was associated with a significant reduction in TG compared to the control group
(Jacobo-Cejudo et al. 2017)	Randomised, single-blind, placebo-controlled pilot study	54	Mexican adults with type 2 diabetes	520mg/d FO for 24 weeks or a placebo	Blood glucose and TG were significantly reduced in the FO group compared to placebo. Placebo group experienced a significant increase in TC and in non-HDL cholesterol. No significant difference between FO and placebo groups were observed for HbA1c and insulin.
(Jamilian et al. 2018)	RCT<br><br>(Randomised, double-blinded, placebo-controlled trial)	40	Women with gestational diabetes	2000mg/d FO for 6 weeks	Gene expression for LDL receptor, IL-1 and TNF-alpha was downregulated following FO supplementation.<br><br>FO supplementation led to a significant reduction in TG and hs-CRP, and a significant increase in LDL and HDL, compared to placebo.
(Karakas et al. 2016)	Randomized, 3-parallel arm, double-blinded study	51	Women with polycystic ovary syndrome	3.5g/d FO, flaxseed oil or soybean oil for 6 weeks	FO and SBO increased insulin response and resistance.
(Kuszewski et al. 2020)	RCT	152	Older sedentary overweight or obese adults	2400mg/d FO for 16 weeks	FO supplementation significantly decreased TG and increased HDL

	(Randomised, double-blinded, placebo-controlled trial)			(2000mg DHA + 400mg EPA)	
(Logan and Spriet 2015)	RCT (single-blinded manner)	24	Older adult females<br><br>(Median age 66y)	3g/d FO for 12 weeks	FO consumption significantly lowered TG levels and placebo group did not. There were no significant differences between changes in CRP or insulin between groups.
(Mansoori et al. 2015)	RCT<br><br>(randomised, double-blind, placebo-controlled clinical trial)	72	Patients with type 2 diabetes	2400mg/d FO for 8 weeks<br><br>(1450mg DHA + 400mg EPA)	FO supplementation was associated with a significant reduction in TG, compared to placebo. There were no significant differences between fasting serum insulin, HcA1c, LDL, HDL or TC between groups.
(Muldoon et al. 2016)	Double-blind, placebo-controlled clinical trial	261	Healthy volunteers	1400mg/d FO for 18 weeks	FO supplementation did not significantly alter serum levels of CRP or IL-6 and no significant differences were found between the treatment and placebo groups.
(Rahmani et al. 2018)	RCT<br><br>(randomized, double-blind, placebo-controlled trial)	40	Women with polycystic ovary syndrome	2000mg/d FO for 12 weeks	FO supplementation significantly reduced the. expression of IL1 compared to placebo. No significant difference in LDL receptor or TNF-alpha was observed between groups.
(Raygan et al. 2019)	RCT<br><br>(randomized, double-blinded,	90	Diabetic patients with coronary heart disease	1000mg/d FO or 1000mg/d flaxseed oil	Both FO and flaxseed oil resulted in a significant reduction in insulin. A significant reduction in hs-CRP was

	placebo-controlled trial)				observed for flaxseed oil but not for FO.
(Shen et al. 2017)	RCT<br><br>(randomized, double-blinded, placebo-controlled trial)	97	Older adults with hypertension and/or hyper-cholesterolemia	1000mg/d FO for 12 weeks.	FO supplementation significantly reduced diastolic blood pressure compared to the placebo.<br><br>Both FO and placebo (soybean oil) significantly decreased TC and LDL and significantly increased HDL, but no significant difference between the groups was observed.
(Skulas-Ray et al. 2015)	RCT	115	Healthy volunteers	0, 300, 600, 900, or 1800mg/d for five months	Increased FO supplementation resulted in significantly increased blood DPA levels which, in turn, resulted in significantly decreased CRP and TG.
(Souza et al. 2020)	Clinical trial	32	Overweight or obese adults with type 2 diabetes for 1 year and hyper-triglyceridaemia	4g/d FO for 8 weeks	TNF-alpha, IL1 and IL6 were significantly decreased and insulin sensitivity was significantly improved following FO supplementation.
(Sparkes et al. 2018)	RCT<br><br>(randomised, double-blind, placebo-controlled trial)	53	Healthy pre-menopausal women with mildly elevated TG	0, 0.35, 0.7 or 1g/d FO for 8 weeks	1g/d FO supplementation reduced TG, but lower doses did not.<br><br>Higher FO dose is significantly associated with increased HDL particle size.
(Suzumura et al. 2016)	RCT	32	Breast cancer patients at the beginning of chemotherapy	4g/d FO for 60 days.	HDL levels in FO group remained the same but were significantly decreased in the control group.

(Toupchian et al. 2016)	RCT<br><br>(double-blind, randomised controlled trial)	72	Type 2 diabetes patients	2.4g/d FO for 8 weeks	No significant change in insulin resistance was observed following FO supplementation.
(Toupchian et al. 2018)	RCT<br><br>(randomized, double-blind, placebo-controlled clinical trial)	72	Type 2 diabetes patients	2.4g/d FO for 8 weeks.	Short-term FO supplementation did not result in a significant change in TNF-alpha and or IL-6 RNA expression.
(Yang et al. 2019)	RCT<br><br>(double-blind, randomized controlled trial)	108	Volunteers with abdominal obesity	2g/d FO (DHA + EPA), 2.5g/d flaxseed oil (ALA) or a corn oil control, for 12 weeks	FO significantly decreased LDL compared to CO, whereas flaxseed oil did not. No significant difference for any outcome was seen between FO and flaxseed oil groups.
(Yang et al. 2020a)	RCT	77	Middle aged or elderly hypertensive volunteers	2g/d FO for 90 days or a corn oil control	Significant reduction in TNF-alpha and CRP levels were observed in the FO group, but no change in IL-6 was observed.
(Yang et al. 2020b)	RCT<br><br>(double-blind, randomised cross-over study)	30	Normolipidemic adults	12g/d of either a DHA- or EPA-rich FO for 8 weeks	A significant reduction in TG was seen in both groups and no changes in cholesterol-related parameters were observed for either group.
(Yang et al. 2020c)	RCT	30	Normolipidemic adults	12g/d saury oil (2.5g omega-11	Saury oil resulted in a significant reduction in LDL compared to

	(double-blind, randomized, crossover clinical trial)			LCMUFA and 3.4g omega-3) or a control (4.9g shorter chain MUFA and 3g omega-3).	control and a minor but significant increase in HDL particle size. No significant differences between groups were observed for TG,
(Zibaeenezhad et al. 2017a)	Randomised, open labelled trial	95	Hyperlipidaemic patients	2g/d FO for 8 weeks or 250g fresh fish twice weekly.	A significant decrease in TC, non-HDL cholesterol and TG was observed for both groups, with HDL being significantly increased in both groups. These changes were less pronounced in the FO group.<br><br>LDL increased in the FO group whilst it was significantly reduced in the fresh fish group.
(Zulyniak et al. 2016)	Placebo-controlled study	32	Healthy young (n = 15) and older (n = 17) men	2g/d EPA and 1g/d DHA for 3 months	FO supplementation resulted in a significant decrease in TG in both young and old men.
